# Maize cytolines as models to study the impact of different cytoplasms on gene expression under heat stress conditions

**DOI:** 10.1186/s12870-022-04023-8

**Published:** 2023-01-02

**Authors:** Ioana V. Ardelean, Loredana Bălăcescu, Oana Sicora, Ovidiu Bălăcescu, Lia Mladin, Voichița Haș, Mihai Miclăuș

**Affiliations:** 1 Biological Research Center, “Babeș-Bolyai” University, Jibou, Romania; 2 NIRDBS, Institute of Biological Research, Cluj-Napoca, Romania; 3 The Oncology Institute “Prof Dr Ion Chiricuta”, Cluj-Napoca, Romania; 4 Agricultural Research and Development Station, Turda, Romania; 5 STAR-UBB, “Babeș-Bolyai” University, Cluj-Napoca, Romania

**Keywords:** Heat-shock response, Gene expression profiling, Organelles, Retrograde signaling, *Zea mays*

## Abstract

**Background:**

Crops are under constant pressure due to global warming, which unfolds at a much faster pace than their ability to adapt through evolution. Agronomic traits are linked to cytoplasmic-nuclear genome interactions. It thus becomes important to understand the influence exerted by the organelles on gene expression under heat stress conditions and profit from the available genetic diversity. Maize (*Zea mays*) cytolines allow us to investigate how the gene expression changes under heat stress conditions in three different cytoplasmic environments, but each having the same nucleus. Analyzing retrograde signaling in such an experimental set-up has never been done before. Here, we quantified the response of three cytolines to heat stress as differentially expressed genes (DEGs), and studied gene expression patterns in the context of existing polymorphism in their organellar genomes.

**Results:**

Our study unveils a plethora of new genes and GO terms that are differentially expressed or enriched, respectively, in response to heat stress. We report 19,600 DEGs as responding to heat stress (out of 30,331 analyzed), which significantly enrich 164 GO biological processes, 30 GO molecular functions, and 83 GO cell components. Our approach allowed for the discovery of a significant number of DEGs and GO terms that are not common in the three cytolines and could therefore be linked to retrograde signaling. Filtering for DEGs with a fold regulation > 2 (absolute values) that are exclusive to just one of the cytolines, we find a total of 391 up- and down-DEGs. Similarly, there are 19 GO terms with a fold enrichment > 2 that are cytoline-specific. Using GBS data we report contrasting differences in the number of DEGs and GO terms in each cytoline, which correlate with the genetic distances between the mitochondrial genomes (but not chloroplast) and the original nuclei of the cytolines, respectively.

**Conclusions:**

The experimental design used here adds a new facet to the paradigm used to explain how gene expression changes in response to heat stress, capturing the influence exerted by different organelles upon one nucleus rather than investigating the response of several nuclei in their innate cytoplasmic environments.

**Supplementary Information:**

The online version contains supplementary material available at 10.1186/s12870-022-04023-8.

## Background

Global warming has a significant impact on crops, with strong reductions in yield being already predicted [1, 2]. The low natural variation of crops makes evolutionary adaptation highly unlikely, with climate change calculated to be 5000-fold faster than the grasses’ ability to change their niche [3]. On a global scale, it is predicted that a 1 °C increase in temperature will lead to a 7% loss in yield for maize (*Zea mays ssp. mays*) [4], one of the most important crops of the *Poaceae* family.

Domesticated about 9000 years ago [5], maize has been under constant human selection. Breeding programs nowadays are faced with an ever-shrinking gene pool [6] due to the use of elite inbred lines for the creation of hybrids, especially after World War II, while the others were discarded. As many inbred lines were left behind, deemed to be unimportant at that time, the maize crop was faced with a major bottleneck in allelic richness, making it vulnerable to global warming.

Agronomic traits and phenotypic variations are linked to cytoplasmic-nuclear genome interactions in a wide range of species, from grasses to mammals, yeast, or *Drosophila* [7–10]. Both mitochondria and chloroplasts play important roles in the plant response to heat stress [11, 12]. The organelles exert their influence on the nucleus through retrograde signals, first discovered decades ago both in the chloroplast [13] and in the mitochondrion [14], and receiving much attention ever since (comprehensively reviewed in [15–18]). Numerous genes influenced by retrograde signaling have already been identified. For example, Gläßer et al., 2014 [19] reported more than 4895 genes that responded to the plastid-to-nucleus signaling through mutation experiments, whereas Schwarzländer et al., 2012 [20] identified over 6500 genes that responded to different chemical treatments in impaired *Arabidopsis* mitochondria and are therefore likely part of retrograde signaling.

**Fig. 1:**
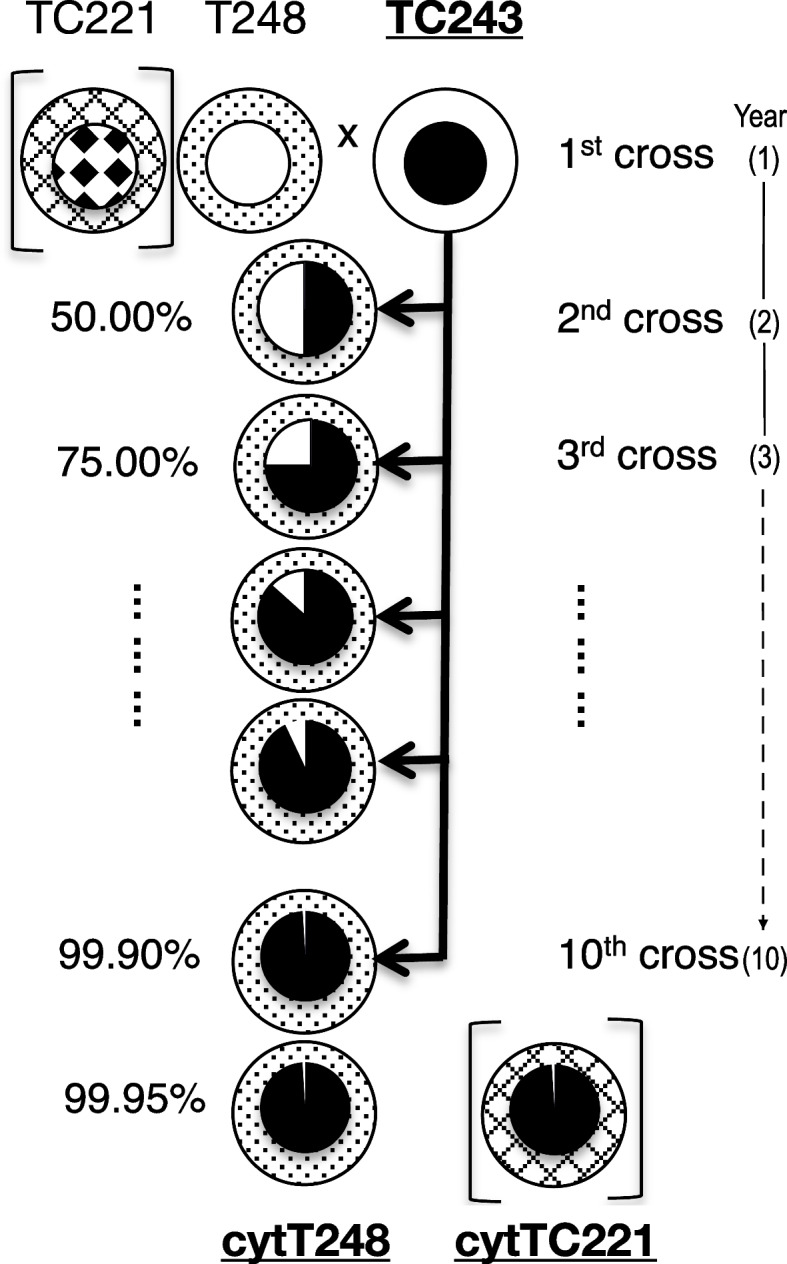
Crossing scheme used to create the three cytolines. TC243 inbred line was back-crossed 10 times, for the duration of 10 years, always as a pollen donor, with TC221 and T248. At the end of the 10^th^ cross, which correspond to the 10^th^ year of the crossing scheme, 99.95% of the acceptor lines/cytoplasm donors’ genetic material is replaced by that of the inbred line TC243. Neither mitochondria nor chloroplasts are transmitted through pollen, resulting in cytT248, cytTC221, and TC243, which share the same nucleus but a different organellar set

Here, we use a different approach in understanding the effect of retrograde signaling, through the use of cytolines under heat stress. As we have previously shown, cytolines are created by at least 10 crosses (Fig. 1, adapted from [21]). The starting inbred lines were chosen to represent three of the major maize genepools world-wide: Iowa Stiff Stalk Synthetic (inbred line TC243), European Flint (inbred line TC221), and Lancaster (inbred line T248). The inbred line TC243 was backcrossed 10 times (always as a pollen donor) to the inbred lines TC221 and T248, respectively. Considering the temperate-continental climate of Romania, which allows for just one maize generation a year, the backcrossing took 10 years to complete. Upon completion of the crossing scheme 99.95% of the acceptor lines/cytoplasm donors’ nuclear genetic material was replaced by that of the inbred line TC243. The resulting three cytolines (TC243, cytTC221, and cytT248) (or isonuclear lines) are inbred lines having the same nuclear DNA, but different cytoplasms, each having its distinct organellar set, exerting their influence on nuclear gene expression. Although leakage has been reported in plants, the mitochondria and chloroplast are not transmitted through pollen in maize [22–24]. Therefore, cytolines offer a unique experimental set-up to address the effect of the cytoplasm, with its constitutive organellar set, on gene expression. Nowadays’ technologies facilitate the study of how plants respond to heat stress from an *omics* perspective, with an atlas of gene expression responses having been developed for various tissues of maize [25], for example.

Complementary to testing the response to heat stress of a single nucleus hosted in a single cytoplasm (as in He et al., 2019 [25]), here, we use three maize cytolines to *(i)* probe how one nucleus performs under heat stress in different cytoplasmic environments (with their distinct organelles) and *(ii)* find a link between gene expression patterns, genetic variability of the organellar genomes and that of the cytolines’ original nuclei. We use a custom-made microarray chip to identify differentially expressed genes (DEGs) at 24 h of stress at 40 °C in 3 weeks old seedlings, and genotyping-by-sequencing (GBS) to genotype the organellar genomes, and the original nuclei that had been replaced in the two cytoplasm donors.

By corroborating gene expression data with the existing polymorphisms in nuclear and organellar genomes, and measurements of photosynthesis rates in the three cytolines, our results offer a unique perspective on the existing interplay between one nucleus and three different cytoplasms. GO enrichment analyses reveal the biological processes, molecular functions, and cellular components that are common, and those that are cytoplasm-specific. Our results contribute molecular data to the paradigm used to explain the anterograde-retrograde cross-talk in a novel experimental set-up that emphasizes the response of one nucleus in the presence of different organelles, and heat stress.

## Results

### One nucleus – three cytoplasms under heat stress

#### Overview of differentially expressed genes (DEGs)

**Fig. 2:**
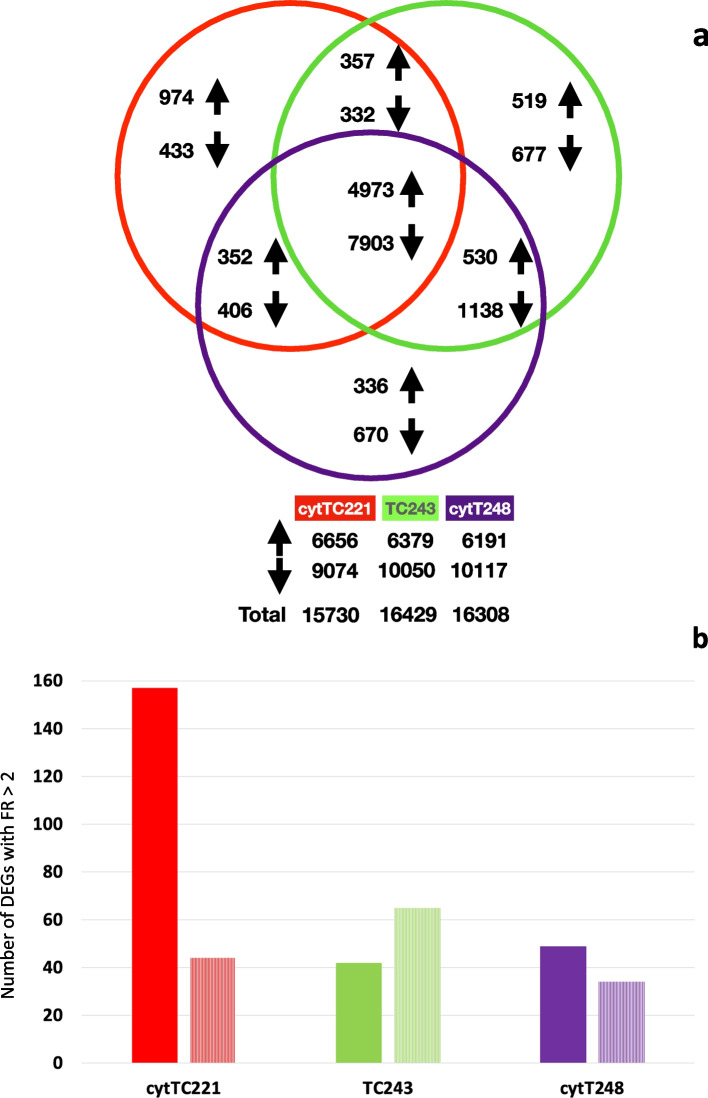
Differentially expressed genes in the three cytolines. *a)* Venn diagram of the up/down-regulated genes in the three cytolines in response to heat stress, symbolized with up/down arrows. *b)* Cytoline-specific up- (solid fill) and down-regulated (pattern fill) genes with a fold regulation > 2 in the three cytolines

We measured the gene expression response to heat stress in three cytolines (cytT248, cytTC221, and TC243) (Fig. 1) and identified 19,600 DEGs. In general, there is a downregulation of genes in response to heat stress, with ~ 6000 up- and ~ 10,000 down-regulated genes per cytoline. The common response of the three cytolines includes 4973 upregulated and 7903 downregulated genes, with heat shock protein (HSP) genes being the most upregulated (Fig. 2a and Additional file 1). Of the 4973 common up-DEGs, 2759 have an FR (fold regulation) > 2 (i.e., more than half of total). There are 17 genes with an FR > 100 in all three cytolines, of which eight are HSPs, and one is a heat shock factor (HSF). Further, 38 have an FR > 50, 146 have an FR > 20, and 413 have an FR > 10.

There are 3312 down-DEGs with an absolute FR value > 2 common in the three cytolines, which accounts for less than half of the reported 7903; only three have an FR > 100 in all three cytolines, 16 with an FR > 50, 91 with an FR > 20, and 280 with an FR > 10 (all FR values are absolute).

#### Cytoline-specific DEGs

As the same nucleus now resides in three different cytoplasms, one would expect the three nucleus-organellar set combinations to influence gene expression accordingly. These influences would reflect in cytoline-specific responses. Of the 19,600 DEGs in our experiment, cytTC221 has the most inbred line-specific (i.e., 974 up- and 433 down-DEGs) (Fig. 2a). The 974 up-DEGs in cytTC221 significantly contrast with the only 519 up-DEGs specific to TC243, and 336 in cytT248, respectively. Also, there is an inverse response in terms of directionality, cytTC221 having more up- than down-DEGs, unlike TC243 and cytT248 where the majority are down-regulated.

Filtering only for cytoline-specific DEGs with an FR > 2 (absolute values) we find a total of 391 up- and down-DEGs. Of these, 204 do not have a functional annotation. The characteristic response of cytTC221 is emphasized by number of DEGs with an FR > 2, this cytoline maintaining almost three times as many up-DEGs compared to either of the other two lines (Fig. 2b). However, the up−/down-DEGs ratio is inversed in case of cytT248 (compared to that from Fig. 2a, where all DEGs are considered), conferring a distinct pattern to the two cytoplasm donor lines compared to TC243, the nucleus donor.

#### Grouping cytoline-specific DEGs into GO terms

We further grouped the cytoline-specific DEGs (i.e., those responding to heat in one of the cytolines, but not in the other two) with an FR > 2 into biological processes, molecular functions, and cell components GO terms (Additional file 2): *(i)* Cytoline-specific DEGs mostly group within GO biological processes associated to transcription, translation, phosphorylation, and protein degradation. *(ii)* Enzymatic functions are the most abundant GO molecular function terms, with 165 records out of the 269. Transferase activity is the molecular function ranking first in terms of number of cytoline specific DEGs grouped within, followed by hydrolase, oxidoreductase and kinases activities. Equally important are the molecular functions associated to binding (mostly nucleic acid or ion binding), present in 76 out of the 269 GO terms described. *(iii)* The cytoline-specific DEGs’ proteins do not localize in a particular GO cell component, being targeted to different cell compartments, mostly to the nucleus, seconded by the membrane.

#### Expression levels of regulatory gene families

**Fig. 3:**
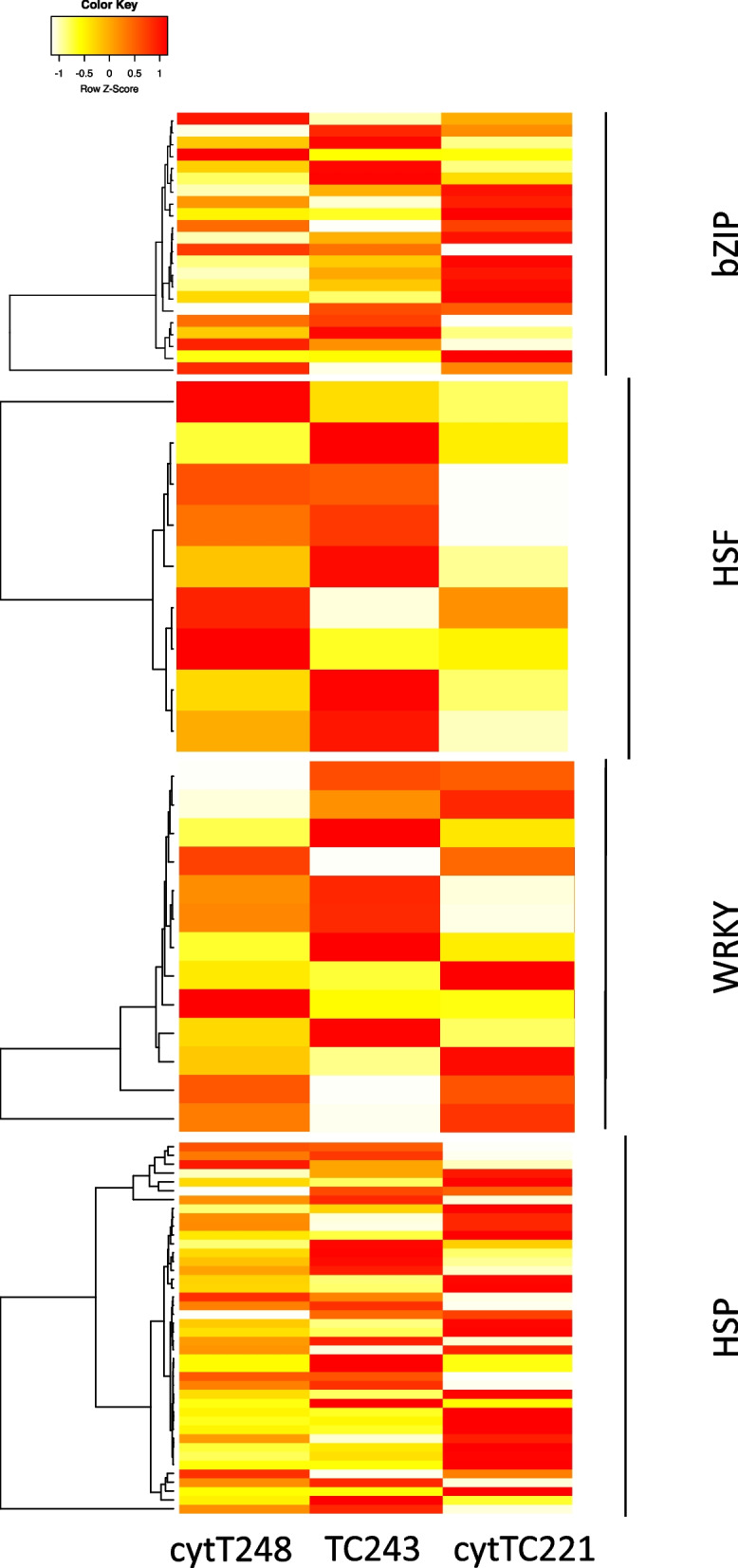
Different expression patterns for the members of the bZIP, HSF, WRKY, and HSP gene families, plotted as heatmaps of the row Z-score. For clarity, only DEGs with and FR > 2 are plotted

Among the gene families that are known to be activated by heat stress signals [26–29] we identified 89 bZIP-, 19 heat stress-, and 77 WRKY-transcription factors as DEGs (Additional file 3). The majority of these family members are common to the three cytolines and include the *bZIP-transcription factor 71* (fold regulation (FR) > 50), *HSF-transcription factor 7* (FR > 125), and *WRKY-transcription factor 78* (FR > 17). Severely down-regulated members of these families include the *bZIP-transcription factor 50* (FR < − 14), *HSF-transcription factor 28* (FR < − 6), and *WRKY-transcription factor 111* (FR < − 20). Among the heat shock proteins (HSP) controlled by these transcription factors, we identified 56, including *heat shock protein26*, with an FR > 500 in all three samples, being the most upregulated gene within the DEGs (Additional file 3). The common DEGs in all of the four gene families (bZIP, HSF, WRKY, HSP) show significant differences in the magnitude of their FR per cytolines, as seen on the heatmaps of the up-DEGs members, with an FR > 2 (Fig. 3).

### GO terms enrichment analyses

To understand the three cytolines’ overall response to heat stress, we performed GO enrichment analyses for *(i) Biological processes*, *(ii) Molecular functions,* and *(iii) Cell components* using all the up- and down-DEGs in each of the three cytolines as input and applying a false discovery rate (FDR) threshold of < 0.05.

#### Enrichment in up-DEGs

*(i) Biological processes.* There are 164 GO biological processes enriched with various fold enrichment (FE) values in all three cytolines (Additional file 4). We chose an FE > 2 threshold to underline the most responsive biological processes to heat stress, and identified 11 biological processes commonly enriched in the three cytolines (Table 1). Nevertheless, this value is arbitrary, as all the 164 GO terms have an FDR < 0.05 in all the three cytolines.

**Table 1:** GO terms enriched with an FE > 2, using up-DEGs as input

GO	cytTC221	cytT248	TC243
**Biological process**			
protein complex oligomerization (GO:0051259)	✓	✓	✓
chaperone cofactor-dependent protein refolding (GO:0051085)	✓	✓	✓
de novo’ posttranslational protein folding (GO:0051084)	✓	✓	✓
protein refolding (GO:0042026)	✓	✓	✓
de novo’ protein folding (GO:0006458)	✓	✓	✓
protein folding (GO:0006457)	✓	✓	✓
response to heat (GO:0009408)	✓	✓	✓
response to salt stress (GO:0009651)	✓	✓	✓
protein localization to endoplasmic reticulum (GO:0070972)	✓	✓	✓
chaperone-mediated protein folding (GO:0061077)	✓	✓	✓
response to osmotic stress (GO:0006970)	✓	✓	✓
protein import into mitochondrial matrix (GO:0030150)	✓		
protein targeting to mitochondrion (GO:0006626)	✓		
protein localization to mitochondrion (GO:0070585)	✓		
establishment of protein localization to mitochondrion (GO:0072655)	✓		
response to organonitrogen compound (GO:0010243)	✓		
cytoplasmic translation (GO:0002181)	✓		
tRNA aminoacylation (GO:0043039)	✓		
amino acid activation (GO:0043038)	✓		
translational elongation (GO:0006414)	✓		
mitochondrial transport (GO:0006839)	✓		
maintenance of protein localization in organelle (GO:0072595)		✓	
cellular response to topologically incorrect protein (GO:0035967)			✓
response to topologically incorrect protein (GO:0035966)			✓
intracellular protein transmembrane transport (GO:0065002)			✓
arginine metabolic process (GO:0006525)		✓	✓
proteasomal ubiquitin-independent protein catabolic process (GO:0010499)	✓		✓
ubiquitin-dependent ERAD pathway (GO:0030433)	✓		✓
ERAD pathway (GO:0036503)	✓		✓
protein transmembrane transport (GO:0071806)	✓		✓
maintenance of protein localization in endoplasmic reticulum (GO:0035437)	✓	✓	
**Molecular function**			
unfolded protein binding (GO:0051082)	✓	✓	✓
Protein self-association (GO:0043621)		✓	
chaperone binding (GO:0051087)	✓	✓	
**Cellular component**			
proteasome complex (GO:0000502)	✓	✓	✓
endopeptidase complex (GO:1905369)	✓	✓	✓
Sm-like protein family complex (GO:0120114)	✓	✓	✓
spliceosomal snRNP complex (GO:0097525)	✓	✓	✓
small nuclear ribonucleoprotein complex (GO:0030532)	✓	✓	✓
U2 snRNP (GO:0005686)	✓		
organellar ribosome (GO:0000313)	✓		
transcription elongation factor complex (GO:0008023)		✓	
catalytic step 2 spliceosome (GO:0071013)			✓
proton-transporting two-sector ATPase complex (GO:0016469)	✓	✓	
proteasome core complex (GO:0005839)	✓		✓

This is a subset of the most enriched GO terms in up-DEGs in the three cytolines, highlighting the common ones in the top tiers of each of the three GO term categories (biologocal process, molecular function, and cellular component). A tick marks the respective GO term as enriched with and FE > 2 in the corresponding cytoline.

To identify biological processes that were likely under retrograde signaling control, we searched for GO terms enriched with FE > 2 in only one of the three cytolines, reporting 10 in cytTC221, one in cytT248, and three in TC243. Equally important are the biological processes enriched with an FE ~ 2 or above in two of the cytolines, but not in the third one, as this indicates they are also part of retrograde signaling (Additional file 4). Thusly, we identified a total of six GO biological process as missing from: cytTC221 (one), cytT248 (four), and TC243 (one) (Table 1).

*(ii) Molecular functions.* There are 30 GO molecular functions enriched with various FE values in all three cytolines (Additional file 4), of which one is commonly enriched with and FE > 2 in the three cytolines, whereas two are enriched either in just one or two cytolines (Table 1).

*(iii) Cell components.* There are 83 GO cell components enriched with various FE values in all three cytolines (Additional file 4). Applying the same FE > 2 filter, we report five cell components as common for the three cytolines, two exclusively found in cytTC221, and one exclusive to either cytT248 or TC243. In addition, one GO cell component is enriched only in the two cytoplasm donor lines, and a second only in cytTC221 and TC243 (Table 1).

#### Enrichment in down-DEGs

We also investigated the enrichment of GO terms using as input the down-DEGs in each of the three cytolines. Unlike the enrichment in up-DEGs, there was no GO term with and FE > 2 for any of the three categories considered (i.e., biological processes, molecular functions, and cell components). However, we report 34 enriched GO terms, most of which are involved in phosphorus metabolism (four biological processes and four molecular functions) or that are part of the chloroplast (nine GO cellular components) (Additional file 4).

**Fig. 4:**
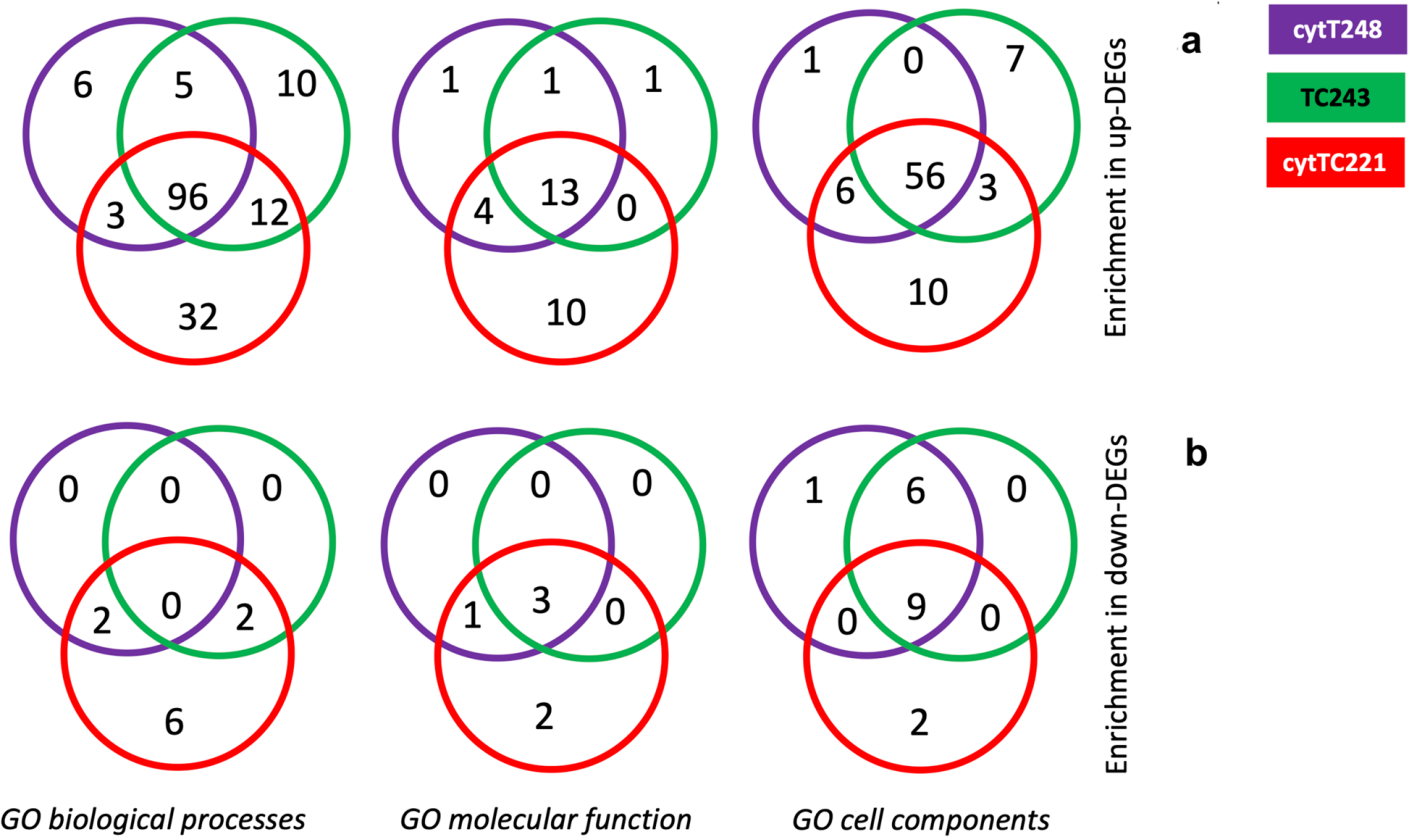
Venn diagrams with counts of enriched GO terms. The number of enriched GO terms in the three cytolines is shown using either their complete set of *(a)* up-, or *(b)* down-regulated genes as input

Overall, when using all the up- and down-DEGs in the three cytolines, *GO biological processes* were the most statistically enriched terms, seconded by *GO cell components* and *GO molecular function*, all with FDR values < 0.05 (Fig. 4 and Additional file 4).

#### The distinct response of cytTC221

When up-DEGs are used as input, cytTC221 stands out with its 32 *GO biological process*, 10 *GO molecular function*, and 10 *GO cell component* terms that are cytoline-specific (Fig. 4a). Although no GO terms are enriched with an FE > 2 when down-DEGs are used as input, cytTC221 has a similar distinct pattern, having 6 *GO biological processes*, 2 *GO molecular function*, and 2 *GO cell component* terms that are cytoline specific, whereas the other two lines have none (Fig. 4b).

### Genotyping the original nuclear and organellar genomes of the three cytolines

**Fig. 5:**
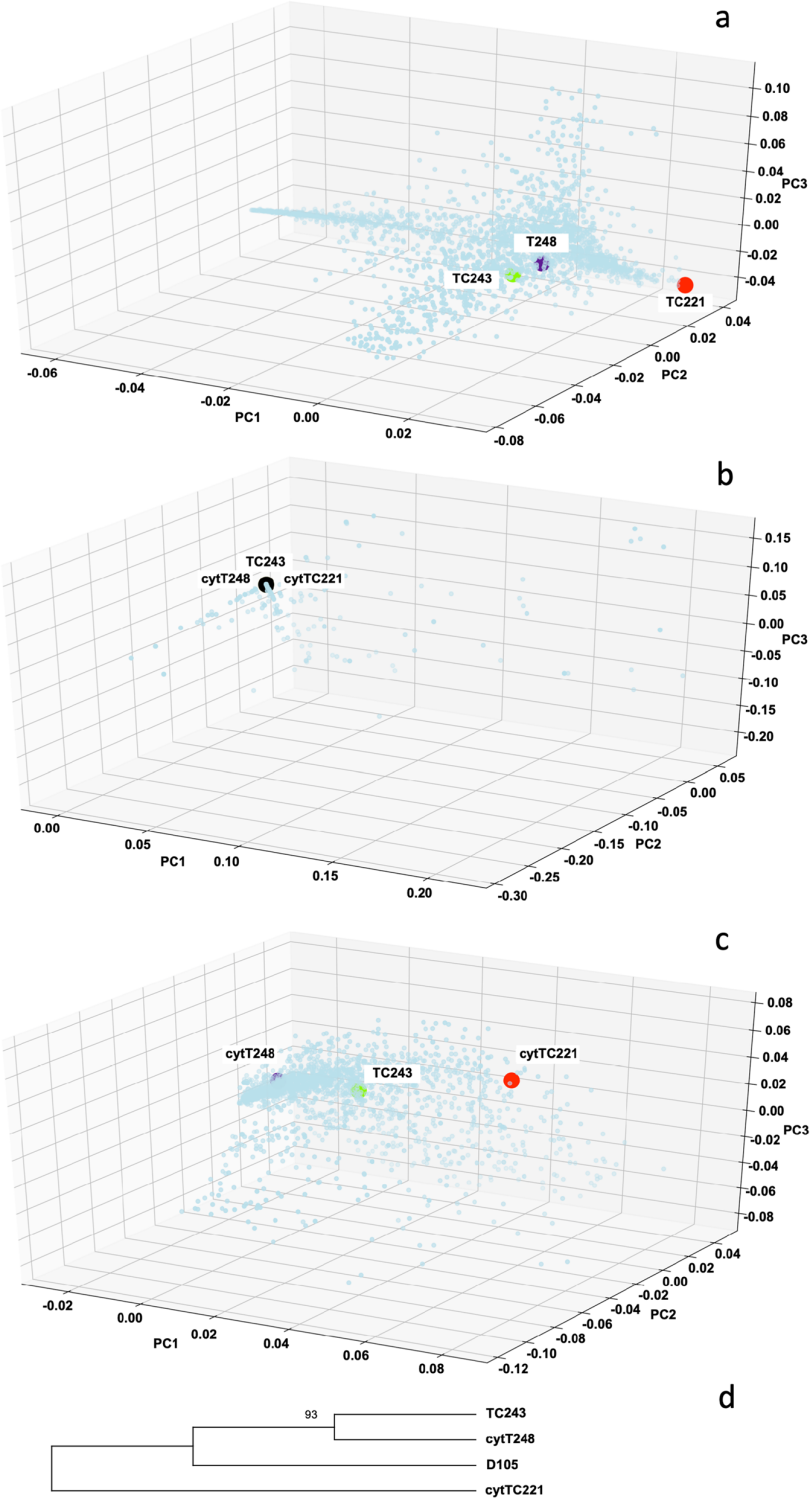
Principal component analysis of the genetic variation in the nuclear *(a)*, chloroplast *(b)*, and mitochondrial *(c)* genomes of 2236 maize inbred lines. *(d)* Divergence of the cytoline mitochondrial genomes calculated using deep-sequencing data is presented as a Maximum Likelihood tree, using inbred line D105 as outgroup

To explain the differences in gene expression found between the three cytolines and the distinct gene expression pattern of cytTC221, we analyzed the variation in the original nuclei of the cytolines and their organellar genomes (which are the same in the inbred lines and in the cytolines, as they are inherited maternally). TC243 (nucleus donor for the present study) and the two cytoplasm donor lines (TC221 and T248) were genotyped in a different study within a group of 2236 inbred lines (unpublished data), TC221 being the most diverged among all (Fig. 5a). On average, more than 390,000 unique reads (> 100 bp) were generated per inbred line, and a mean of ~ 269,000. The organellar genomes of the 2236 inbred lines were also genotyped with 23 chloroplast markers and 80 mitochondrial markers (Fig. 5b,c). In general, there is little variation in the chloroplast genomes of the 2236 inbred lines, the three inbred lines having an identical genetic background at the studied loci. Conversely, the mitochondrial markers differentiate the three inbred lines, the mitochondria of TC221 being the most divergent on the PC1 axis, but not to the extreme shown by its original nucleus.

To confirm the genotyping data of the organellar genomes, generated by skim sequencing, we deep sequenced the mitochondrion and chloroplast of the three cytolines generating more than 500,000 unique reads (> 100 bp) per cytoline and included an outgroup inbred line, D105. We were thus able to identify 527 mitochondrial and 48 chloroplast markers, respectively, for which we had sequencing data in all four samples. Similar to the results obtained with skim sequence data, we observed no variation of the plastid markers in the three cytolines, whereas the mitochondrial genome of cytTC221 diverged from the other two cytolines (Fig. 5d). To further investigate this divergence, we intersected the SNP positions of the 527 markers with the GFF gene description file available for the maize mitochondria [30] and concluded that most of the mitochondrial SNPs are located in intergenic regions or hypothetical proteins, only 133 markers being within genes (Additional file 5). As all of the 133 intragenic markers are identical among the cytolines, the cytTC221 divergence is explained by the remaining intergenic markers or those that are part of genes coding for hypothetical proteins.

### Different patterns of expression for the Pentatricopeptide repeat proteins (PPR)

Despite all intragenic SNPs being identical among the cytolines, the 3D structure of the organellar-encoded proteins may significantly vary *in vivo* due to the gene editing activity of the PPR family of genes. We therefore investigated the gene expression response of 495 PPR genes in our cytolines under heat stress, being particularly interested in the PPR-PLS subfamily and its subgroups, E1, E2, E+, and DYW, as these are known to include most of the editing PPR proteins (reviewed in [31]). We identified 128 PPR-PLS as differentially expressed in at least one of the three cytolines (Additional file 6). The common response is composed of 29 up- and 36 down-DEGs. There is a stark difference between TC243, cytT248, and cytTC221 in the number of cytoline-specific PPRs that are up/down-regulated, the last cytoline standing out with its 13 line-specific up-DEG-PPRs, compared to only one, and two, respectively; a pattern that is reversed for the down-DEG-PPRs, where cytTC221 has the least number of differentially expressed PPR genes.

### The impact of heat stress on photosynthesis

**Fig. 6:**
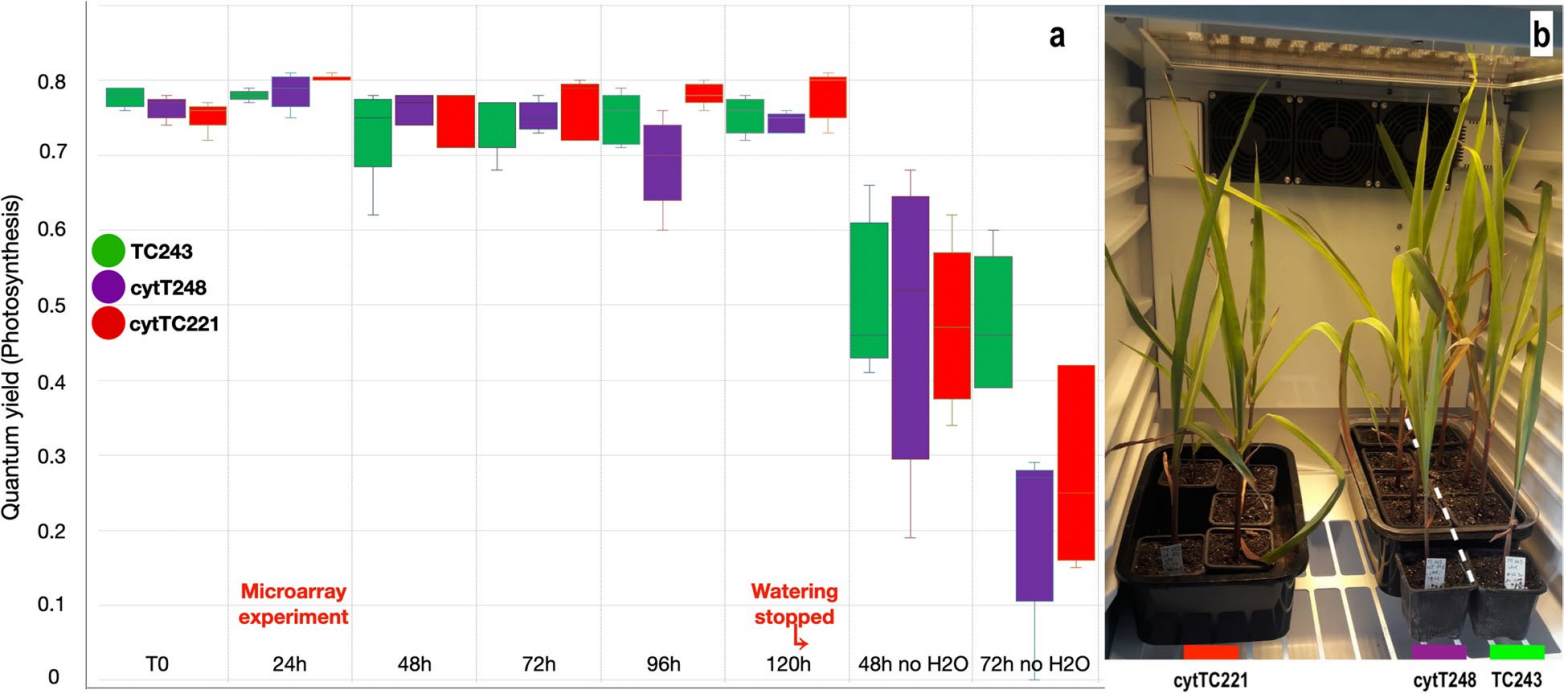
Photosynthesis efficiency *(a)* and growth state of plants *(b)* in the nucleus donor line (TC243) and the two cytoplasm donors (cytTC221 and cytT248) under constant heat stress (40 °C) and daily watering until 120 h, which is the timepoint for the picture in panel *b*. Heat stress continued for 72 h more, but the watering stopped. The timing of the microarray analysis is shown. Marked on the boxplots are error bars and the mean value

The photosynthesis rate was measured in all three cytolines during normal watering and for 72 h after the last watering (Fig. 6a). Heat stress alone did not induce a significant change in photosynthesis efficiency, cytTC221 having a slightly increased rate compared to the other two cytolines, quantum yield (Qy) values being ~ 0.8. However, prolonged heat stress corroborated with hydric stress, severely affected all three cytolines. At 72 h after the last watering and at 192 h after heat stress had started, TC243 had a mean Qy value of approx. 0.5, whereas photosynthesis in the cytoplasm donor lines dropped to a mean of < 0.3.

Photosynthesis related genes encoded by the nuclear genome are generally downregulated (Additional file 7). Similarly, of the 53 DEGs in the chloroplast genome, 46 are down-regulated (Additional file 1, tab six, bottom tier). Unlike the common response of the mitochondrial genes in the three cytolines, which have a similar up regulated FR value, the chloroplast common DEGs are less down regulated in the two cytoplasm donor lines compared to their nucleus donor line (heatmaps included in Additional file 1, tab six, bottom tier).

## Discussion

### DEGs involved in heat stress response, from a retrograde signaling perspective

Although retrograde signaling in plants has been investigated before by various methods (reviewed in [32, 33]) placing one nucleus in three different cytoplasmic environments to study the impact of retrograde signaling on gene expression under heat stress conditions has never been done before. Consequently, our study unveils a plethora of genes that are part of the plant’s response to heat, and that are attributable to retrograde signals.

Out of the total 19,600 DEGs described here, we report 6724 DEGs that are potentially part of the retrograde signaling process in response to heat stress (Additional file 1), being differentially expressed either in one (3609) or two of the cytolines (3115), but not in the other two, or the third one, respectively. As the nuclear genome is the same in all three cytolines, we can speculate that these genes are influenced by the organellar set in which the nucleus currently resides.

The sum of 3609 genes differentially expressed in just one of the cytolines is composed of 1829 up- and 1780 down-DEGs (Fig. 2a and Additional file 1). They underline the importance of anterograde-retrograde signaling, and its control of gene expression in the cell during heat stress. Further functional analyses of these genes may enlarge the existing body of data describing the plant response to heat stress, from a retrograde signaling perspective. Of particular interest for such future studies are the 204 DEGs we report as having an FR > 2 (absolute values) and which lack a functional annotation (Additional file 1).

In addition to the 204 unannotated genes likely involved in retrograde signaling, our results confirm and broaden previous studies by adding new functional data to already annotated genes. It is beyond the scope of the present study to review the extant functional annotations of all DEGs identified here as cytoline-specific, but choosing the three most up-regulated genes in the three cytolines offers a perspective of how our data enlarge their functional annotation: *(i) GRF-transcription factor 6* (Zm00001d017742, FR = 27, the highest FR for cytT248-specific DEGs) has been described before in *Arabidopsis* as negatively regulating leaf growth and restricting cell proliferation in leaf primordia [34]. Another study [35] had reported that norflurazon, which inhibits retrograde signaling and chloroplast differentiation, has the same effect on the plant phenotype as the *grf-transcription factor 6* mutant. Thus, our data confirms and corroborates the findings of the previous two studies, validating Zm00001d017742 as part of retrograde signaling [34, 35]. *(ii) Citrate synthase1* (Zm00001d015856, FR = 21, the highest FR for TC243-specific DEGs) has been reported before by several studies as being part of retrograde signaling in yeast, reviewed in [36], but our data confirms it for the first time as being part of retrograde signaling in plants. *(iii) Actin3* (Zm00001d035720, FR = 9, the highest FR for cytTC221-specific DEGs). Actin bundles, which are part of cytoskeleton, are omnipresent in the plant kingdom, but their biological role is still poorly understood [37]. As ACTIN3 is a structural constituent of cytoskeleton, our data bring new insight into the gene’s functioning by placing its expression under the influence of retrograde signals, which has not been reported before. Furthermore, *actin depolymerizing factor* (ADF) is involved in the plant’s response to abiotic stress [38]. Indeed, we report *severe depolymerization of actin1* gene (Zm00001d002190) as up-regulated exclusively in cytTC221.

While the high FR values for some of the genes are a good indication of their importance in cell signaling under heat stress, their grouping into GO terms offers a broader perspective.

### GO terms involved in heat stress response, from a retrograde signaling perspective

In a meta-analysis in *Arabidopsis*, comprising six different experiments Gläßer et al., 2014 [19] report 461 unique GO terms triggered by retrograde-signaling, most of which are involved in transcription and translation. Unlike our experimental set-up, all six experiments rely on chemical treatments or mutations to induce a retrograde signaling response, which still are the two most used approaches. Our results confirm 56 of the reported GO terms, being enriched selectively, either exclusively in one of the cytolines, or in two, but not common to all. However, we identified 83 new GO terms, in addition to those reported for *Arabidopsis*. Filtering the 83 GO terms for an FE > 2, our study adds 17 GO terms to the paradigm used to explain retrograde signaling: *(i) Biological processes* - maintenance of protein localization in endoplasmic reticulum (GO:0035437), proteasomal ubiquitin-independent protein catabolic process (GO:0010499), protein import into mitochondrial matrix (GO:0030150), ubiquitin-dependent ERAD pathway (GO:0030433), protein targeting to mitochondrion (GO:0006626), protein localization to mitochondrion (GO:0070585), establishment of protein localization to mitochondrion (GO:0072655), ERAD pathway (GO:0036503), response to organonitrogen compound (GO:0010243), cytoplasmic translation (GO:0002181), translational elongation (GO:0006414), mitochondrial transport (GO:0006839), protein transmembrane transport (GO:0071806); *(ii) Molecular functions* – chaperone binding (GO:0051087); and *(iii) Cellular components* - proteasome core complex (GO:0005839), U2 snRNP (GO:0005686), proton-transporting two-sector ATPase complex (GO:0016469).

Interestingly, of the 17 GO terms, the most enriched is common to the two cytoplasm donor lines - maintenance of protein localization in endoplasmic reticulum (GO:0035437), FE ~ 4 in both cytolines, and not the nucleus donor cytoline. The high fold enrichment of GO:0035437 in the two cytolines only, corroborated with the significant enrichment of GO terms such as protein refolding (GO:0042026), de novo’ protein folding (GO:0006458), protein folding (GO:0006457), which are common to the three lines, confirm that the unfolded protein response (UPR) is triggered under heat stress, as it has been shown before including for maize [39]. But there seems to be a retrograde signaling component in what GO terms involved in UPR are most enriched in DEGs, and possibly a time-dependent response of each of the cytolines, as our data capture gene response at a single timepoint after heat stress (24 h).

The distinctive response of cytTC221 in terms of biological processes exclusively enriched with and FE > 2 in this line hints towards a retrograde control of the process described in yeast and humans as MAGIC (mitochondria as guardian in cytosol), important for maintaining the cytosolic proteostasis under heat stress [40]. MAGIC postulates that cytosolic aggregates are imported into the mitochondria for degradation. Indeed, the biological processes exclusively enriched in cytTC221, which are related to the mitochondria (protein import into mitochondrial matrix, protein targeting to mitochondrion, protein localization to mitochondrion, establishment of protein localization to mitochondrion, and mitochondrial transport) (Table 1), are arguments to consider the process as taking place in this cytoline at 24 h of heat stress. Furthermore, the GO biological process maintenance of protein localization in organelle is enriched with an FE > 2 exclusively in cytT248.

Based on all of the above, we can only speculate that both UPR and MAGIC have a regulating component that is part of retrograde signaling, but further research is needed.

Most importantly, all of the above retrograde signaling effects on gene expression and enrichment of GO terms may be caused by the intergenic SNPs in the mitochondrial genomes, as the intragenic SNPs and those on the chloroplast are identical among the three cytolines, and the nuclear genome is identical.

### DEGs – common response

As previously reported, most of the up-DEGs under heat stress conditions are either HSPs, molecular chaperones stabilizing and helping refold heat-inactivated proteins, or transcription factors (reviewed in [41, 42]). Our experiment fits within such conditions, sampling the RNA in maize seedlings at 24 h after heat exposure. Consequently, we report the HSP family members to be significantly up-regulated, including the most up-regulated DEG (*heat shock protein26*, with an FR > 500 in all three samples).

HSP generation is under the influence of HSF, bZIP, WRKY, and MBF1C (Multiprotein-Bridging Factor 1C) transcription factors [26–29], all of which confer resistance to plants under heat stress conditions (reviewed in [43, 44]). Although there is a strong common response in terms of DEGs in all of the four gene families (Additional file3) and MBF1C (Additional file 1), we describe contrasting patterns in the magnitude of gene expression response, measured as FR values (Fig. 3), possibly caused by the different retrograde signals present in the three genetic backgrounds.

Transcription factors in general, not just the families described above, are one of the three layers of the paradigm used to describe retrograde signaling, together with the signals generated at organellar level, and their transducers towards the nucleus (reviewed in [45]). Indeed, among the 19,600 DEGs we report 1406 transcription factors (Additional file 1). Of importance for future studies are the 254 genes, which are specific to one of the three cytolines, an indication for their involvement in retrograde signaling. Following functional analyses, some of the 254 transcription factors described here may enlarge the list of only eight such genes proven to be part of retrograde signaling [45].

### GO terms – common response

Similar to DEGs responding to stress, many studies have focused on identifying the GO terms enriched in such genes. An extensive review by Janni et al., 2020 [42], grouped the enriched GO terms from the existing literature in crop plants and revealed dramatic differences between species, as shown by comparing heat stress response in tomato and maize. However, the comparison rendered some common enriched GO terms, largely confirmed by our results, and which are very likely to be conserved among species. For example, protein folding (GO:0006457) is strongly enriched in tomato and rice, and response to heat (GO:0009408) is strongly enriched in maize and tomato [42, 46], our results confirming them as enriched with and FE > 2 in all three cytolines.

The GO terms we report here as enriched in DEGs and that form the common response of the three cytolines complement existing similar studies in maize [44, 47] adding new biological processes, cellular components, and molecular functions that play a role in heat stress response.

For example, Frey et al., 2015 [47], investigated the response of European maize inbred lines to heat and found 26 and nine GO terms enriched in up- and down-DEGs, respectively. Comparatively, we report 164 and 12 GO terms enriched in up- and down-DEGs, respectively, confirming 10 GO terms enriched in up-DEGs reported by Frey et al., 2015 (GO:0006457, GO:0034641, GO:0044238, GO:0005976, GO:0044264, GO:0044042, GO:0006073, GO:0005576, GO:0048046, GO:0005576) (Additional file 4). Three possible explanations for the significantly higher number of enriched GO terms in our analyses, and for the relatively low overlap with previous data reside in: *1)* the experimental set-up with cytolines, which is able to unmask otherwise elusive GO terms, when different nuclei are probed in their innate cytoplasms. Here, we have one nucleus that respond to three cytoplasms; *2)* the continuous development of the GO terms database [48], which had not been available when Frey et al., 2015 [47] published their findings; and *3)* the different temperature treatment, which was long-time exposure to mild (32/27^o^ C day/night) or strong heat (38/33^o^ C day/night) levels, rather than heat-stress at 40^o^ C for 24 h, as here.

Furthermore, similar to Shi et al., 2017 [44], who report pathways that are kept in balance under heat stress, being enriched in both up- and down-DEGs, we report GO terms with the same pattern in at least one of the three cytolines: *(i)* two *biological processes* (protein folding (GO:0006457) and gene expression (GO:0010467)); *(ii)* three *molecular functions* (transferase activity, transferring phosphorus-containing groups (GO:0016772), kinase activity (GO:0016301), and phosphotransferase activity, alcohol group as acceptor (GO:0016773)); *(iii)* and five *cellular components* (ribonucleoprotein complex (GO:1990904), plastid (GO:0009536), chloroplast (GO:0009507), plasma membrane (GO:0005886), extracellular region (GO:0005576)).

The role of mitochondria in the stress response is substantiated by the six significantly enriched GO cellular components in up-DEGs that are mitochondrion-specific in all of the three cytolines: mitochondrial matrix (GO:0005759); mitochondrial protein-containing complex (GO:0098798); mitochondrial inner membrane (GO:0005743); mitochondrial envelope (GO:0005740); mitochondrial membrane (GO:0031966); mitochondrion (GO:0005739), (Additional file 4), in addition to the more general ones, like “organelle” or “organelle envelope”. Conversely, there is no chloroplast specific GO cellular component enriched in up-DEGs. Instead, nuclear down-DEGs, significantly enrich the chloroplast-related GO cellular components, none being mitochondrion-related (Additional file 4). This is not surprising, as the chloroplast is known to be sensitive to heat stress, corroborated with a decrease in photosynthesis [11].

### The nuclear and organellar genomes

Using our GBS data with inbred lines, we show that the original nucleus of cytTC221 is the most divergent of the > 2300 inbred lines genotyped (Fig. 5a). Its mitochondrial genome exhibits the same divergence (Fig. 5c). It’s arguable that the observed sequence variation in the mitochondrial genomes is influenced by NUMTs (nuclear copies of mtDNA), which have been described on the long arm of maize chromosome 9. Their sequence identity with the mitochondrial genome ranges between 93.81 and 100% [49]. Nevertheless, the influence of these nuclear genomic parts should be minimal, as the organellar genomes out-number the nuclear genome by as much as 5000 to 1 [50, 51] and most of the sequencing data is consequently of organellar origin. Additionally, we confirmed the genetic distances between the mitochondria of the three cytolines using additional deep sequencing data (Fig. 5d).

Considering the genetic distance between the inbred lines, backcrossing TC243 to TC221 could be similar to an interspecific cross. Indeed, working with wheat lines and its closest relatives, Crosatti et al., 2013 [52] showed that gene expression and metabolism are significantly impacted under alloplasmic conditions, with the greatest changes resulting from the widest crosses. Furthermore, Noyszewski et al., 2014 [53], showed that there was more change in the mitochondrial genome of *Triticum*-*Aegilops* alloplasmic lines in a timespan of half a century than there was between *T. turgidum* and *T. aestivum*, during 100 centuries of evolution. Similarly, in the case of our two cytoplasm donor lines, the mitochondrial genome of cytTC221 could have accumulated more mutations than cytT248 (all in the intergenic space) during the 10 years of backcrossing and subsequent self-cross for maintaining the stock.

### The impact of the mitochondrial genome

From a genomic point of view, the observed variation of gene expression in the three cytolines may be explained by the variation in the mitochondrion genome, as the nuclear genome is identical (i.e., innate to TC243) and the chloroplast genome has no genetic variation at the studied loci (Fig. 5b,c). However, the chloroplast may exert its role in heat stress response through different mechanisms, the three cytolines having common chloroplast-encoded DEGs but different magnitudes of their fold regulation (Additional file 1, tab six). The PPR proteins are equally important, as it has been shown that approximately 50% of the members of the PLS-class, investigated here, are targeted to the chloroplast and only 30% to the mitochondria [54]. Therefore, the PPR-PLS proteins may preferentially exert their editing functions withing the chloroplast, but receiving signals directly from the mitochondria or through the nucleus.

How the mitochondria could influence the chloroplast during heat stress is largely unknown [20, 45]. AOX (alternative oxidase) genes are the most studied indicators of mitochondrial retrograde signaling, which play an important role in efficient cell reprogramming under stress [55], being induced by both mitochondrial and chloroplast perturbation [45], confirming the close relationship between the cellular organelles. Indeed, our dataset confirm the upregulation of all three AOX genes in maize *(Zm00001d002436, Zm00001d002434, Zm00001d002435*), but their expression levels differ among the three cytolines (Additional file 1).

We can speculate that the DEGs we report here in just one or two of the cytolines, and that are important in the chloroplast functioning are targets of the mitochondrial signaling directed at controlling the other organelle. For example, *Zm00001d009589* (light harvesting chlorophyll a/b binding protein3) is significantly down-regulated only in cytTC221 (FR = − 224), and is therefore a good candidate for retrograde signaling resulted from the mitochondrial diversity of this line (Fig. 5c). Other such examples include, *Zm00001d043325* (plastid transcriptionally active chromosome 12 homolog; FR = 2.8), *Zm00001d030638* (photo-system b P domain-containing protein1; FR = 2.1), and *Zm00001d053076* (NAD kinase 2 chloroplastic; FR = 3.5), which are upregulated only in TC243, or *Zm00001d007328* (ABC transporter I family member 11 chloroplastic), which is downregulated only in cytTC221 (FR = − 2.1) and cytT248 (FR = − 3.6).

### Breeding perspective

Transferring the TC243 nucleus into TC221 and T248 cytoplasms significantly improved yield [56], but improving this agronomically important trait comes with a cost when exposed to heat stress and/or drought. As we show in Fig. 6a, the nucleus donor line has an advantage in photosynthesis efficiency over the other two cytolines when heat and water stress are combined. Moreover, stress seems to favor the original nucleus-cytoplasm combination, whereas under normal conditions a heterosis-like effect is visible in cytTC221 and cytT248 for plant height and yield [56, 57], similar to the results from previous studies on hybrids created from genetically distant inbred lines [58].

## Conclusions

Our study adds a new facet to the paradigm used to explain how gene expression changes in response to heat stress, capturing the influence exerted by different organelles upon one nucleus rather than investigating the response of several nuclei in their innate cytoplasmic environments. This approach has not been explored yet, previous experiments relying on the use of chemical treatments or mutations to alter retrograde signaling and observing the effect in the cell. In addition to reporting new DEGs and GO terms that have not been associated before to retrograde signaling when heat stress is present, we confirm and broaden extant data on heat stress response in general, adding new genes and GO terms associated to it. However, a meta-analysis across plant species is needed, as it is impossible to track all the genes that are affected by either retrograde signaling or heat stress, in general, from the literature. Consequently, we see the need of a database to centralize these genes.

From a genomic point of view, the uniqueness of the nuclear genome in the three cytolines, corroborated with the lack of polymorphism in the chloroplast genome, underline the importance of the mitochondria in heat stress response. Furthermore, the mitochondrial polymorphism is part of the intergenic regions and hypothetical genes, rather than genes *per se*.

## Methods

### Plant material and growth conditions

Three seeds from each of TC243, cytTC221, and cytT248 were grown in a Binder KBW 240 growth chamber for 20 days with 16 h of light at 26 °C and 8 h of dark at 20 °C. On the 21st day, the temperature was increased to 40 °C and maintained for 24 h under the same light conditions. The leaves and stems of three seedlings per inbred line (three-leaf stage) were harvested at the end of the heat stress period and flash frozen in liquid nitrogen. Control samples were grown and harvested at the end of the 21st day of normal growth conditions.

To measure photosynthesis efficiency, five seedlings from each of TC243, cytTC221, and cytT248 were grown in a FytoScope Chamber FS 130 (Photon Systems Instruments) up to three-leaf stage (16 h of light at 26 °C and 8 h of dark at 20 °C). Recordings of the Qy (photosystem II Quantum Yield) values were taken at T0 (control temperature) and five more times at 40 °C stress every 24 h, seedlings being watered daily with 30 ml water. No significant differences in the grow state of plants in any of the three cytolines were observed, as shown in Fig. 6b. Following the 5th measurement, the seedlings were not watered anymore, and the temperature was maintained at 40 °C. Two more measurements of Qy were taken at 48 h and 72 h, respectively. A FluorPen FP 100 (Photon Systems Instruments) was used to measure the Qy values for each of the five seedlings and averaged per each of the three lines.

### RNA extraction, microarray assay and data analysis

Total RNA was isolated with TriReagent (Sigma–Aldrich). Four replicates for each genotype-condition were used, under normal and stress temperatures. Total RNAs were further purified with RNeasy Mini Kit (Qiagen) and their quality was evaluated with Bioanalyzer 2100 (Agilent Technologies) based on RNA integrity. RIN (RNA integrity number) was > 8 for all samples. Cy-3 labeled microarray probes (cRNA-Cy3) were synthesized according to Agilent manufacturer’s protocol. The probes were hybridized to Agilent maize custom arrays 4x180k containing 176,026 in situ synthesized 60-mer oligonucleotide features. Preprocessing and differential data analysis were performed on the median signal from raw files generated by the Feature Extraction software v. 11.0, using standard functions in R/Bioconductor (https://www.bioconductor.org) and custom written routines. The differentially expressed sequences were selected with the *limma* package by fitting a linear model for each sequence and using empirical Bayes smoothing to moderate the standard errors. A gene was considered differentially expressed when the *P* value adjusted for multiple testing (Benjamini–Hochberg method) was < 0.01. In total, our microarray platform detected 30,331 of the 39,324 protein coding genes included in the v4 release of the maize reference genome [59]. The MaizeGDB conversion tool [60] was used for converting the v3 gene IDs (which had been spotted on the microarray) to v4. The raw microarray data were deposited in NCBI under GEO accession number GSE171670.

### Bioinformatic pipelines and tools used in data analysis

GeneOntology [48, 61] (http://geneontology.org) was used for GO term enrichment analyses, choosing *Zea mays* as a model species and using a reference gene set composed of all the genes detected by our platform (i.e., 30,331). The cytoline-specific variation in enriched GO terms, was investigated using the differentially expressed genes responding to heat stress in each of the three cytolines. By intersecting the three lists of GO terms, the common and specific responses in the three cytolines were defined. We also used GeneOntology for grouping into GO terms the DEGs exclusively found in one of the three cytolines, having an FR > 2.

Two NGS data sets (i.e., Genotyping-By-Sequencing) were used in our analyses: the first included the original three inbred lines that donated the nucleus and cytoplasm, respectively, whereas the second comprised the three cytolines and an outgroup. For the first, DNA of inbred lines TC243, TC221 and T248, was skim sequenced (data deposited in EMBL under accession number PRJEB43714) together with other 2233 inbred lines from SE Europe (unpublished data). For the second, the organellar DNA of TC243, cytTC221 and cytT248 was deep sequenced (data deposited in EMBL under accession number PRJEB45081). In both cases, total DNA libraries were prepared and sequenced in an Illumina HiSeq X instrument. Raw data were cleaned with default settings in Fastp [62]. Mapping of short reads onto the B73 reference genome, v4 release [59, 63], was done using BWA mem [64]. Mapped reads were processed with SAMtools [65], piping the output to GATK3 (UnifiedGenotyper) [66] for calling the SNPs. BEAGLE 5.0 [67] was used for imputations of the genotyping data, and GCTA [68] for PCA calculations. Plotting the results in 3D was done with *pyplot* and *mpl_toolkits* Matplotlib libraries [69]. To call the SNPs of organellar TC243, cytTC221, and cytT248 genomes, the BAM files resulted from deep sequencing were pooled with the BAM files of the 2233 inbred lines. The SNPs for which we had sequence data in all of the three cytolines (plus an outgroup sample – D105 inbred line) were converted to fasta format and used as input in MEGA X [70] to calculate the best model for building a maximum likelihood (ML) tree. Model T92 was chosen, according to lowest BIC score and the bootstrap consensus tree was inferred from 1000 replicates. All nuclear and organellar SNP markers used in the PCA and ML analyses were defined using the --maf 0.05 and --geno 0.1 parameters in PLINK 1.9 [71, 72], to include only SNPs with a 90% genotyping rate in all samples (except for deep sequencing data, where we considered only the SNPs for which we had data in all samples). Additionally, we pruned the SNP dataset to correct for linkage disequilibrium using the --indep-pairwise 100 5 0.5 parameter, which removes a pair of SNPs if the linkage disequilibrium is greater than 0.5 considering a window of 100 SNPs, and shifting the window 5 SNPs forward before repeating the procedure. The group of --maf, −-geno, and --indep-pairwise parameters implemented in PLINK 1.9 assured a reliable SNP dataset for downstream analyses.

## Supplementary Information

**Additional file 1. **Differentially expressed genes in the three cytolines. Up- and down-DEGs identified in this study are presented in six tabs, as gene IDs, annotations, and fold regulation (FR) values. Various groupings of these DEGs are indicated in the headers. Tabs 1–5 include nuclear DEGs, whereas the 6th tab contains organellar DEGs and associated heatmaps for the mitochondria (upper tier) and chloroplast (lower tier), respectively

**Additional file 2. **Grouping of cytoline-specific DEGs with an FR > 2 into GO terms. Each of the three GO term categories (biological processes, molecular functions, and cell components) has a dedicated tab. Column headers indicate the GO term ID, the dataset considered (i.e., up/down genes exclusively found in one of the cytolines), the total number of genes included in a particular GO term and the GO term’s full name.

**Additional file 3. **Fold regulation values for members of four gene families: bZIP, HSF, WRKY transcription factors, and HSP.

**Additional file 4. **GO terms enrichment analyses. Fold enrichment values of GO terms (biological process, molecular function, cell component) are presented in each of the three cytolines, using up- or down-DEGs as input in the analysis. A tick marks the GO terms that are commonly enriched in all cytolines.

**Additional file 5. **Intersection of mitochondrial SNPs with the available GFF file. The intersect of the 527 mitochondrial SNP markers resulted from deep sequencing with the available GFF file [30] is detailed. The 133 intragenic markers are highlighted in yellow.

**Additional file 6. **Up- and downregulated PPR genes in response to heat stress, grouped as common or cytoline-specific responses in the three cytolines.

**Additional file 7. **Downregulation of photosynthesis-related genes* in the nuclear genome. A tick differentiates the genes that are downregulated in all three cytolines from the ones that are downregulated in at least one. The genes that are greyed out were not differentially expressed in our experiment. *as defined in He et al., 2019 [25].

## Data Availability

Expression data are deposited in NCBI under GEO accession number GSE171670 (https://www.ncbi.nlm.nih.gov/geo/query/acc.cgi?acc=GSE171670). Genomic data are deposited in EMBL under accession numbers PRJEB43714 (https://www.ncbi.nlm.nih.gov/Traces/study/?acc=PRJEB43714%20&o=acc_s%3Aa) and PRJEB45081 (https://www.ncbi.nlm.nih.gov/Traces/study/?acc=PRJEB45081&o=acc_s%3Aa).

## Abbreviations

DEG: differentially expressed gene
FDR: false discovery rate
FE: fold enrichment
FR: fold regulation
GBS: genotyping-by-sequencing
GFF: general feature format
GO: gene ontology
HSF: heat shock transcription factors
HSP: heat shock protein
MAGIC: mitochondria as guardian in cytosol
PLS: one of the two main classes of PPR proteins, contain alternating canonical P-type motifs and variant long (L)- and short (S)-type motifs
SNP: single nucleotide polymorphism
UPR: unfolded protein response
